# Combination therapy with micellarized cyclopamine and temozolomide attenuate glioblastoma growth through Gli1 down-regulation

**DOI:** 10.18632/oncotarget.17205

**Published:** 2017-04-18

**Authors:** Yu-Jie Liu, Ying-Cong Ma, Wen-Jie Zhang, Zhen-Zhen Yang, De-Sheng Liang, Zhi-Fu Wu, Xian-Rong Qi

**Affiliations:** ^1^ Beijing Key Laboratory of Molecular Pharmaceutics and New Drug Delivery System, School of Pharmaceutical Sciences, Peking University, Beijing, 100191, PR China; ^2^ School of Materials Science and Engineering, Baise University, Guangxi Baise, 533000, PR China

**Keywords:** cyclopamine, temozolomide, hedgehog pathway, synergistic effect, glioma

## Abstract

Glioblastoma multiforme (GBM) is the most common and deadly brain cancer, characterized by its aggressive proliferation to adjacent tissue and high recurrence rate. We studied the efficacy and related mechanisms of the combination of cyclopamine (Cyp, a Sonic-hedgehog pathway (Shh) inhibitor) and temozolomide (TMZ, the clinically most used chemotherapeutic agent) in anti-GBM treatment. The micellarized Cyp (MCyp) showed better performance than Cyp solution in inhibiting GBM cells proliferation (3.77-fold against U87 MG cells and 3.28-fold against DBTRG-05MG cells) and clonogenity (1.35-fold against U87 MG cells and 2.17-fold against DBTRG-05MG cells), and preferred behavior of inhibiting cell invasion, colony formation through attenuated Gli1 expression. In addition, combination of MCyp and TMZ exhibited synergistic cytotoxicity, correlating with their ability in inducing apoptosis and eliminating neurospheres formation, and the combination of TMZ was accompanied with the enhanced blockage of Shh pathway. The optimal ratio of MCyp combined to TMZ was 1:20. So we proposed to use TMZ to kill tumor parenchyma and MCyp as the cancer stem cells inhibitor to resist tumor recurrence. These findings demonstrated that combination of TMZ with micellarized Cyp is a promising strategy for exerting different functions of drugs for tumor treatment.

## INTRODUCTION

Glioblastoma (GBM) is one of the most dangerous cancers and is among the most aggressive cancers [1]. GBM cells invade and migrate along white matter tracts [2] and blood vessels [3]. The first choice of GBM treatment is surgical resection. However, due to the complexity and vulnerability of human intracranial structure, as well as the infiltrative nature of GBM cells, a complete removal of GBM cells seems impossible [4]. Without thorough removal, the recurrence of GBM becomes unavoidable which consequently reduces patients’ life expectation to less than one year [5, 6]. As a result, chemotherapy becomes a vital part in GBM treatment. However, traditional drug therapy is significantly impaired by blood-brain barrier (BBB) and efflux transport proteins (P-gp). Therefore, a majority of drugs that show great anti-tumor potential *in vitro*, yield little efficacy *in vivo*. Until now, with the pharmacological intervention, the medium living period of the GBM patients is only 14.6 months.

Temozolomide (TMZ), a DNA-damaging agent, is the first-line chemotherapeutic agent for GBM in clinical practice. The most significant advantage of TMZ is its ability to pass BBB and subsequently to the central nervous system (CNS) and inhibit the growth of GBM cells. However, TMZ causes severe side effects, including myelotoxicity, ulcers, nausea, vomiting, fatigue and headache [7]. Meanwhile, the up-regulation of O-6-methylguanine-DNA methyltransferase (MGMT) in the cells after the treatment of TMZ makes the cancer resistant to it. In addition, the resistance of glioma to TMZ and radiation therapy may be related to the existence of cancer stem cells (CSC) [8–10]. More and more evidence indicates that tumor contains and is caused by CSC which possess the capability of reinitiate tumor development after standard treatment [11]. This means, even though TMZ could inhibit the proliferation and terminate most of the glioma cells, it is still unable to prevent recurrence [12, 13]. Therefore, more effective treatment strategies are needed to achieve the following goals: inhibit the migration, overcome resistance, prevent recurrence and reduce side effects, all of which together will serve to prolong the life expectancy and improve the life quality of patients.

Sonic-hedgehog (Shh) signaling pathway plays significant roles in the early development of CNS. Most high-grade brain tumors, including GBM, overexpress Shh [14]. Shh mediated transduction is activated through the binding of Shh to its receptor Patched (PTCH) [15], which is the repressor of Smoothen (Smo). Smo activation leads to Gli1-dependent transcription factors’ up-regulation through classic Shh pathway, including N-myc, cyclin D, PTCH, Gli1 and Gli2 [16]. It is demonstrated that Shh pathway regulates the expression of stemness genes and the self-renewal of CD133^+^ glioma stem cells [17, 18]. What's more, Gli1 has been shown linked to increased expression of MGMT, which contains a putative Gli1-binding site [19]. Thus, we assumed that Shh pathway inhibitors could enhance TMZ in glioma treatment.

Cyclopamine (Cyp), an effective Shh antagonist, competitively binds to the Smo receptor and inhibits the activation of Shh pathway. It is also selective, which is non-toxic to cell types that are not dependent on the activation of the Shh pathway [20]. Regression of tumors in basal cell carcinoma patients after Cyp treatment were observed in preclinical trial [21]. The poor aqueous solubility of Cyp (< 1 mM) [22] is a major drawback for its clinical development [23]. In addition to the poor solubility, Cyp is also known to be CNS toxic for embryos, resulting in cleft lip and palate in mice [24] and somatic stem cells [25].

The studies of biocompatible poly(ethylene glycol)-block-poly(D, L-lactide) (PEG-PLA) copolymer micelles as a novel drug carrier have become more popular in the current decade [26]. PEG-PLA copolymers have also been approved by Food and Drug Administration (FDA) for multiple applications [27]. There has been an approved product based on PEG-PLA micelles in South Korea for the poorly water-soluble paclitaxel [28]. PEG can provide a hydrated steric barrier which can avoid protein adsorption, recognition and sequestration by the body's defense system-the reticuloendothelial system (RES). PLA could be metabolized to water and carbon dioxide which can be excreted from the body.

In the present study, to take advantage of the benefits of PEG-PLA micelles, we encapsulated Cyp into PEG-PLA micelles. The micellarized Cyp (MCyp) not only improves the solubility of Cyp, but also its cytotoxicity is 3 times that of Cyp. MCyp demonstrates better ability in preventing cells migration and suppressing in colony formation. When MCyp is combined with TMZ, they increase Shh-mediated Gli1 down-regulation. The selectivity of the two drugs directly translates into suppression of tumor growth as a therapeutic outcome in spheroids and neurospheres. Therefore, these results provide a unique strategy to suppress the glioma and improve therapeutic efficacy.

## RESULTS

### Characteristics of MCyp and blank micelles

The critical micelle concentration (CMC) value of polymeric micelles was examined using pyrene, largely employed as hydrophobic fluorescent probe. The plot of the intensity of I_340_/I_337_ ratio of the pyrene against the negative logarithm of the polymer concentration is used for the calculation of CMC. The CMC value of the PEG-PLA copolymer was about 2.16 × 10^−7^ mol/L. The low CMC of the copolymer suggests it has a good thermodynamic stability. Meanwhile, the interaction between the polymer molecules makes it highly dynamically stable [29].

One of the major roles of delivery vehicle is to enhance the solubility of highly lipophilic drugs in an aqueous medium. In our study, the maximum amount of Cyp loaded into 0.1 mL of a 3% (w/w) PEG–PLA micelle solution was found to be 0.15 mg. This amount was equivalent to 1.5 g in 1 L of a 3% (w/w) PEG–PLA micelle solution while the solubility for Cyp in a physiological solution (pH 7.4) is observed less than 30 μM. The PEG–PLA micelles have enhanced the solubility of Cyp in water by a factor of more than 121.

**Figure 1:**
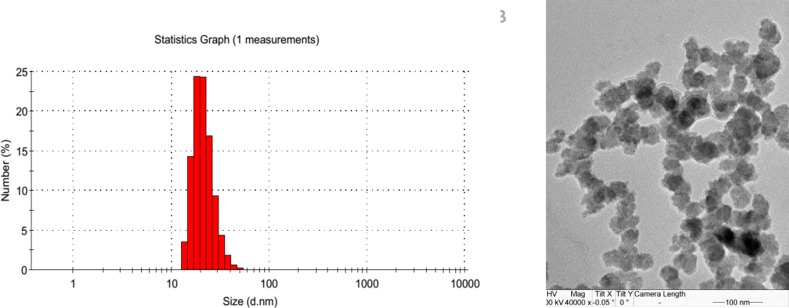
Size of MCyp determined by dynamic light scattering (**A**) and transmission electron microscope (**B**).

The mean diameter of MCyp was about 30 nm by dynamic light scattering (DLS) and transmission electron microscope (TEM) (Figure 1). The small size of the micelles and the PEGylation can take advantage of permeability and retention (EPR) effect, decrease the uptake of RES and prolong circulation time *in vivo* [30]. It is demonstrated that the blood clearance of the small nanoparticles (< 80 nm) was much lower than larger ones (> 200 nm) [31]. As an additional barrier for the nanoparticles access into the brain, the pores in brain extracellular matrix (ECM) possess diameters ranging from 38~64 nm [32] and only nanoparticles below 100 nm is possible to enhance the penetration in brain tissues [33, 34]. The micelles were negatively charged with zeta-potential absolute values of 3~7 mV. Encapsulation efficiencies of these micelles were found to be above 90%. There were no significant differences in size, morphology and zeta-potential between blank micelles and MCyp.

### MCyp inhibit cancer cell proliferation and clonogenicity

**Figure 2:**
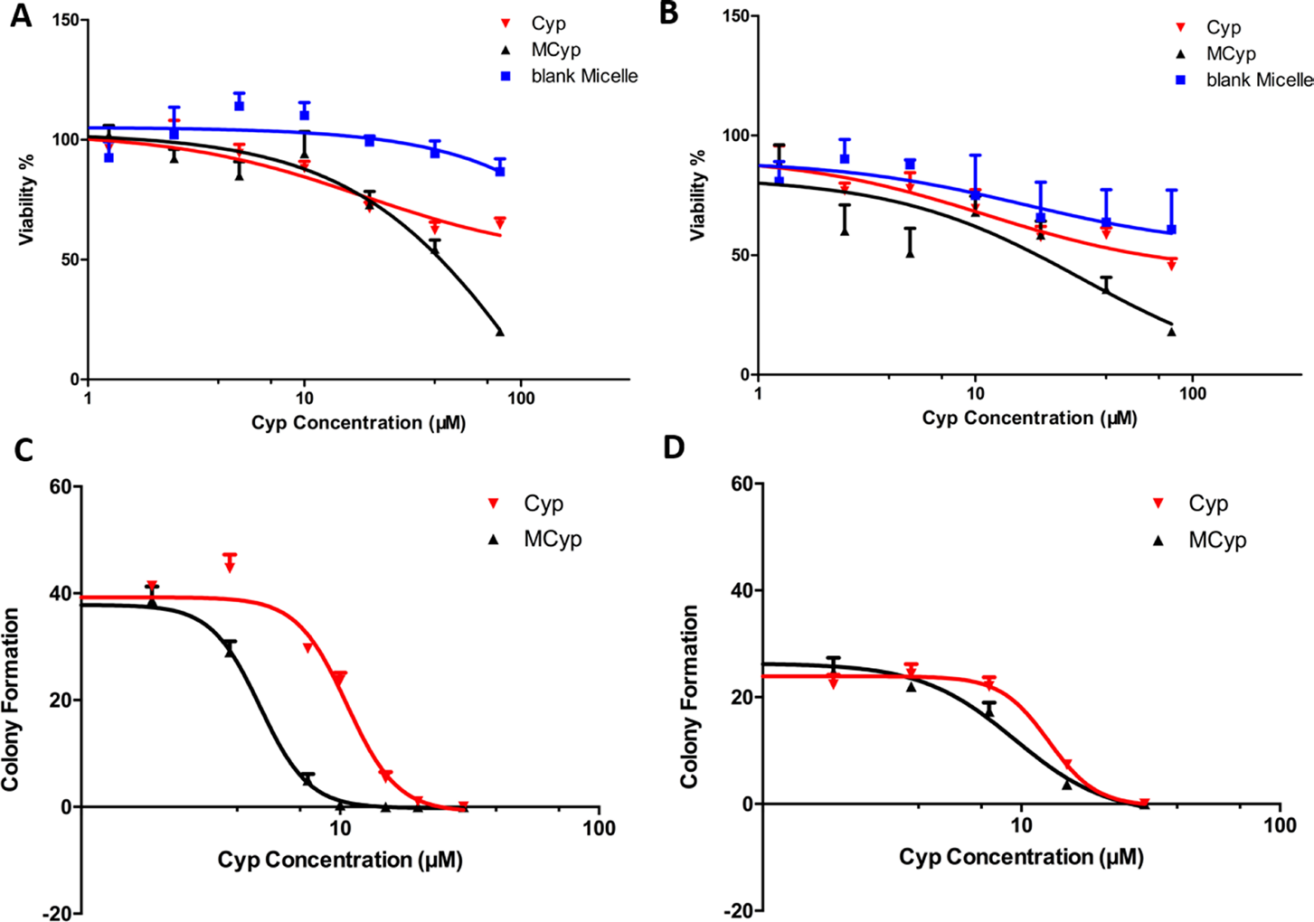
Survival rate of DBTRG-05MG cells (**A**) and U87 MG cells (**B**) cultured with Cyp solution, MCyp and the corresponding blank micelles for 48 h. Data are presented as the mean ± SEM (*n* = 6). Colony formation number of DBTRG-05MG cells (**C**) and U87 MG cells (**D**) after different concentrations of Cyp solution and MCyp treatment. Data are presented as the mean ± SEM (*n* = 3).

MTT assay was conducted to study the cell proliferation inhibition. The results showed that MCyp was more effectual than Cyp solution in inhibiting the proliferation of GBM cells. We chose U87 MG cells and DBTRG-05MG cells for our study. DBTRG-05MG cells were derived from an adult female with glioblastoma multiforme who had been treated with local brain irradiation and multidrug chemotherapy, which should be suitable for the study of glioma therapy. The IC_50_ values of MCyp against DBTRG-05MG cells and U87 MG cells were 35.20 and 14.65 μM, which were reduced by 3.28-fold and 3.77-fold than the values of Cyp solution, respectively (Figure 2A, 2B). The blank micelle did not show significant toxicity to the cells, which guaranteed the safety of the drug carrier for further study.

Colony formation assay is a method to determine a single cell reproductive ability to grow to a colony against treatment [35], and has been used as a standard method *in vitro* to evaluate stem cell-like behavior in both tumor and non-tumor tissues [36]. Both U87 MG cells and DBTRG-05MG cells express CD133^+^ (0.67% and 2.73%, respectively, tested by FACScan flow cytometry after marked by CD133/2-PE). After the cultivation DBTRG-05MG cells and U87 MG cells with different formulations, the number of colonies was counted and EC_50_ was calculated. Results (Figure 2C, 2D) showed that both MCyp and Cyp solution can inhibit the colony formation in both cell lines, and cells seemed more sensitive to MCyp. The EC_50_ of MCyp to DBTRG-05MG and U87 MG cells were 4.904 and 9.427 μM, and the EC_50_ of Cyp solution is 10.62 μM and 12.76 μM in DBTRG-05MG and U87 MG cells, respectively.

### MCyp inhibit cancer cells wound reparation, invasion and migration

**Figure 3:**
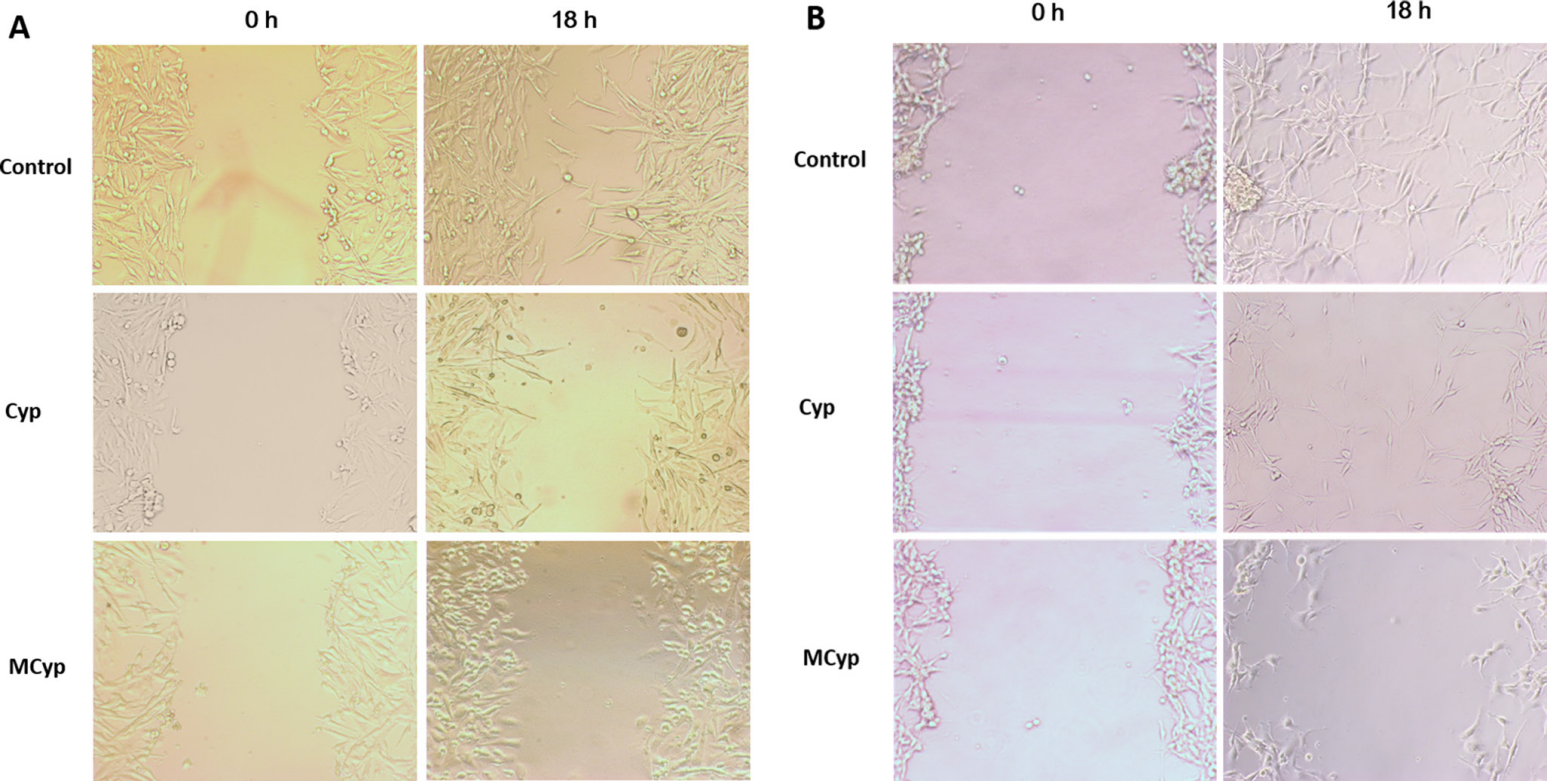
Representative images of the wound healing assay at 0 and 18 h for DBTRG-05MG (**A**) and U87MG (**B**) cells. The final concentration of Cyp was 5 μM.

As the invasive identity of GBM cells prevents thoroughness of excision, the inhibition of migration of the cells is essential in the treatment of glioma. Therefore, MCyp and Cyp solution were assessed by cell scratch test (Figure 3). The wound area in control group repaired notably. The groups of Cyp solution and MCyp barely migrated compared to the control group. Meanwhile, MCyp had stronger inhibition of reparation than Cyp solution in both cell lines.

**Figure 4:**
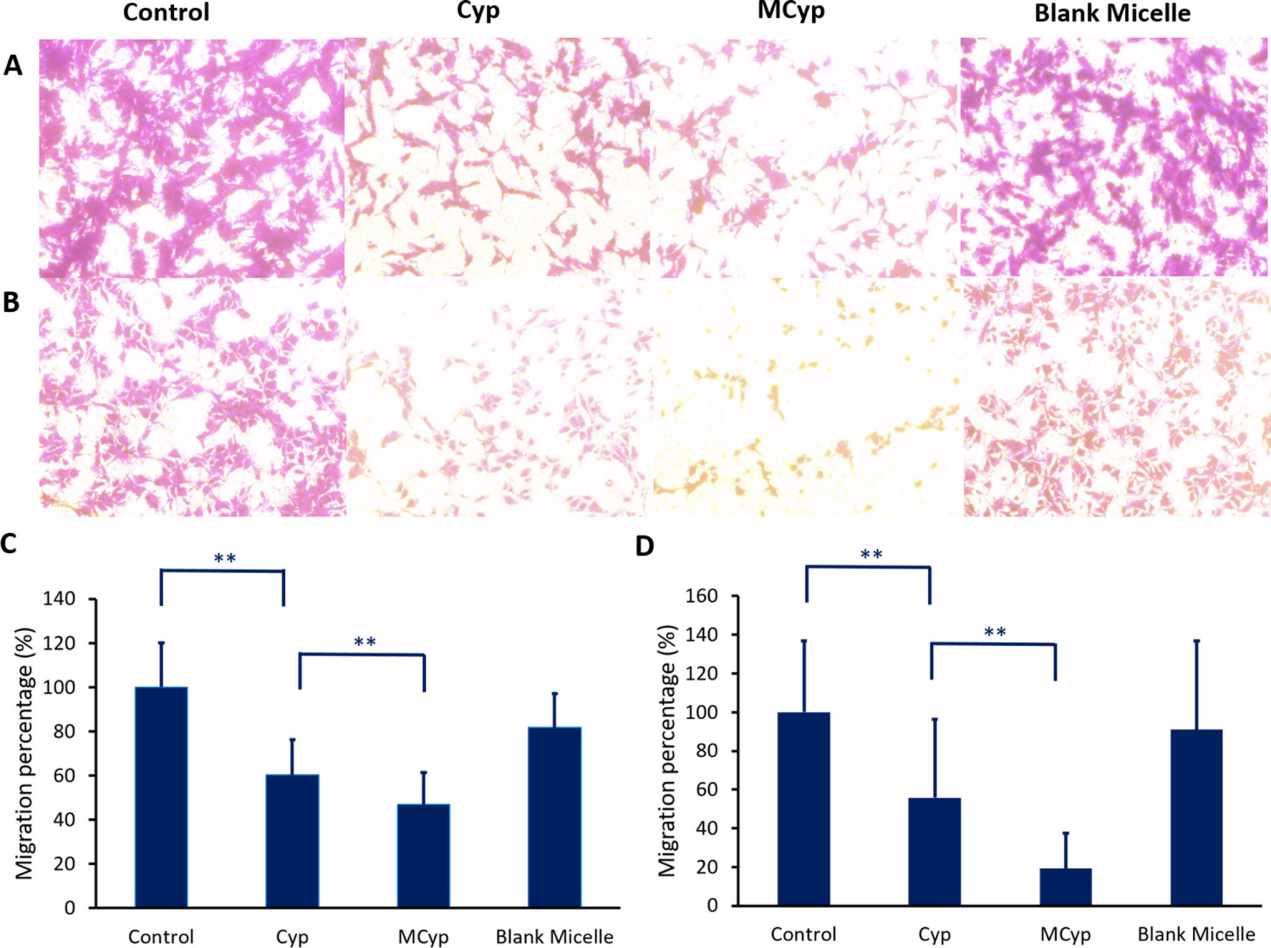
Image of migration of DBTRG-05MG cells (**A**), U87 MG cells (**B**) after incubation with Cyp solution, MCyp and blank micelles for 30 h, respectively (magnification 10×). The final concentration of Cyp was 5 μM. Quantitative analysis of the relative percentage of DBTRG-05MG (**C**) and U87 MG cells (**D**) migrated compared to the control group. Data are presented as the mean ± SD (*n* = 17). Asterisks denote significant *P* < 0.001.

**Figure 5:**
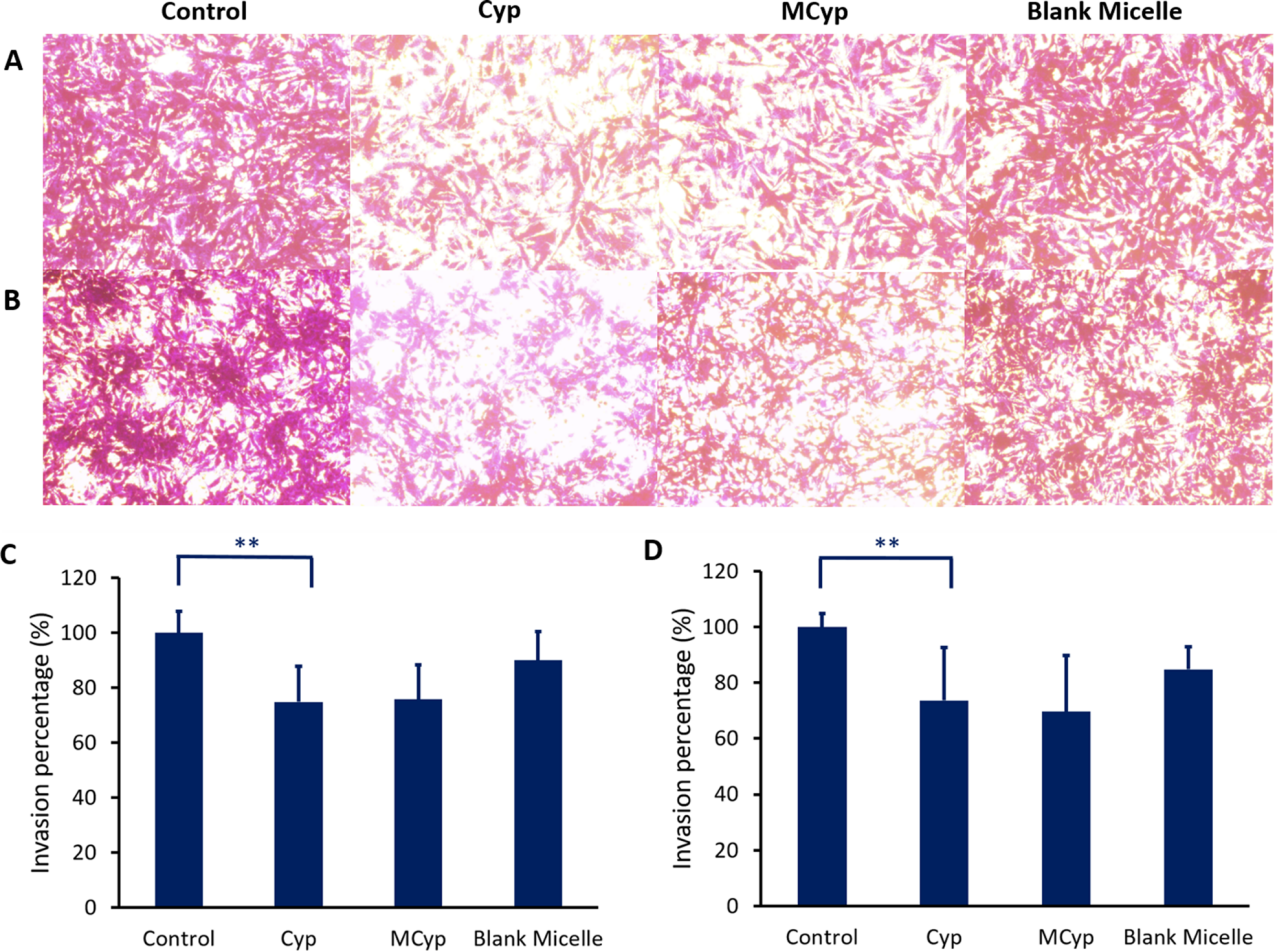
Image of invasion of DBTRG-05MG cells (**A**), U87 MG cells (**B**) after incubation with Cyp solution, MCyp and blank micelles for 30 h, respectively (magnification 10×). The final concentration of Cyp was 5 μM. Quantitative analysis of the relative percentage of DBTRG-05MG (**C**) and U87 MG cells (**D**) invaded compared to the control group. Data are presented as the mean ± SD (*n* = 22). Asterisks denote significant *P* < 0.001.

The migration assay (Figure 4) and cell invasion assay (Figure 5) were carried out in transwell plates, the cells on the back of the membrane were monitored by microscope, which were the migrated cells due to the induction of culture medium. The images were analyzed by MATLAB^®^, and the pixels of which RGB is greater than (250, 250, 250) were calculated (white area), the remaining pixels were determined as the area of cells. MCyp demonstrated remarkably better migration (*P* < 0.001) and invasion inhibition abilities than Cyp in both DBTRG-05MG and U87 MG cells.

### MCyp sensitize the cytotoxicity of TMZ

**Figure 6:**
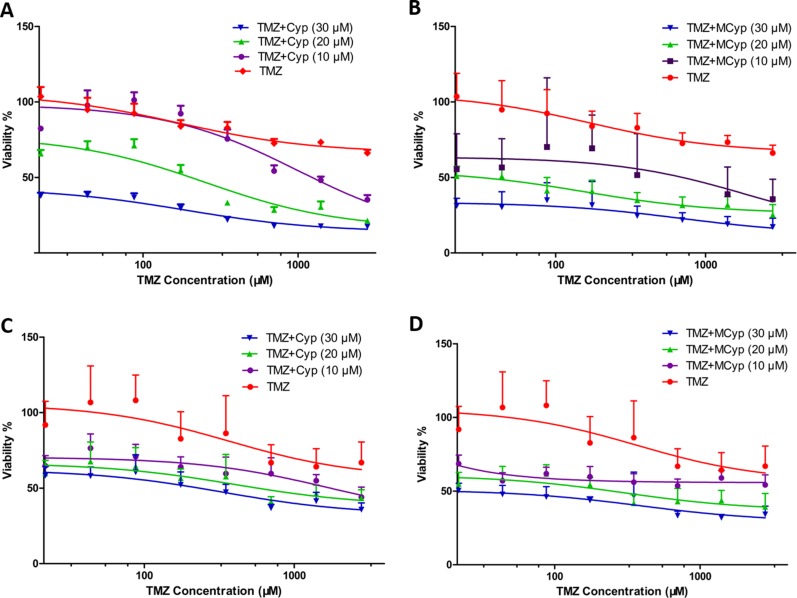
Survival rate of DBTRG-05 MG cells (**A**, **B**) and U87 MG cells (**C**, **D**) cultured with different concentrations of TMZ and fixed concentrations of Cyp solution (A, C) or MCyp (B, D) for 48 h. Data are presented as the mean ± SEM (*n* = 6).

To study whether the presence of micellarized Cyp could enhance the cytotoxicity of TMZ to DBTRG-05 MG cells and U87 MG cells, MTT assay were tested at different concentrations of Cyp combined with TMZ. When TMZ alone is used to treat the cells, the survival rate of the cells did not change much even at high concentrations of TMZ (Figure 6). However, the inhibitory potency was significantly enhanced when certain concentration (10, 20 or 30 μM) of Cyp solution or MCyp were used in combination with TMZ and showed a dose-dependent pattern. Unsurprisingly, MCyp possess better effect in inhibiting cell viability when combined with TMZ than that of Cyp solution. Our results suggest the additive efficacy of MCyp and TMZ in GBM cells is suitable for bulk tumors. The more effective MCyp seems to take more advantage of the micelle formulation.

### Synergistic effect and ratio of TMZ and MCyp in inhibition growth of U87 MG cells

**Figure 7:**
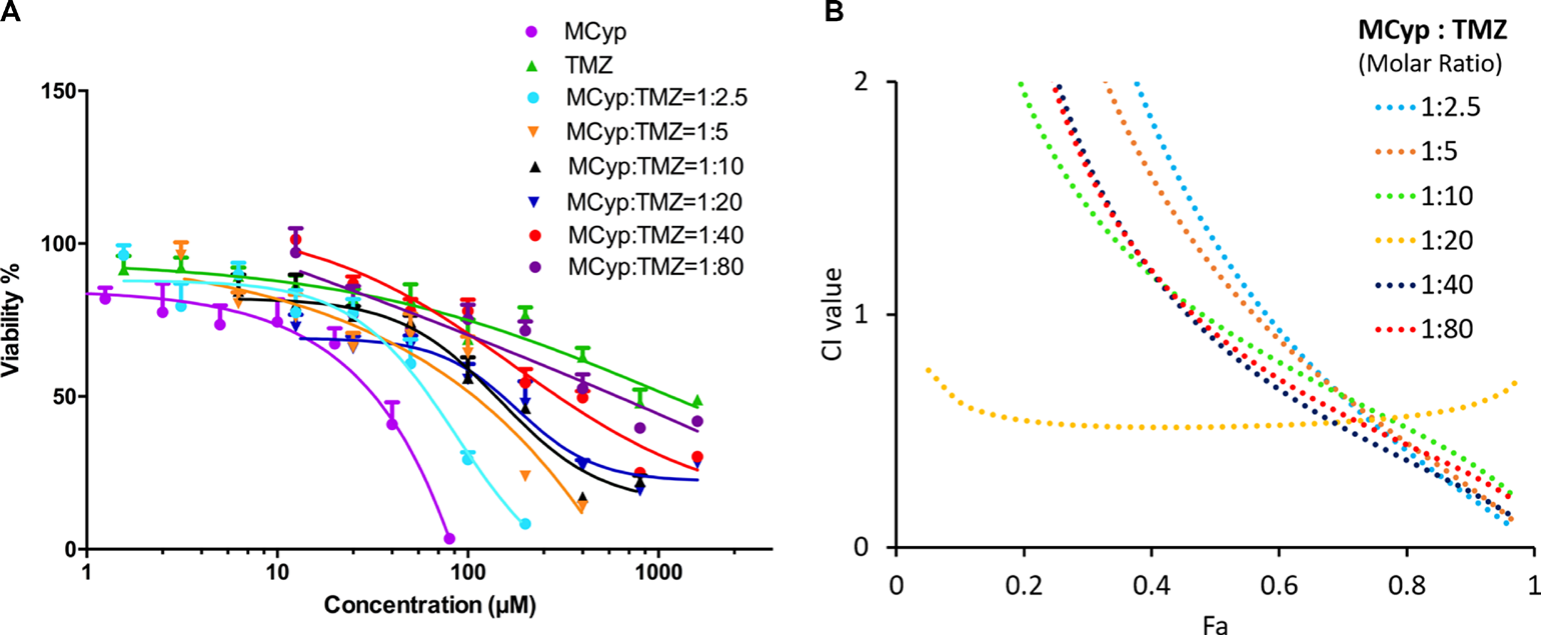
Cytotoxicity study of free TMZ, MCyp and both drug combinations at various molar ratios after 72 h (**A**) and the corresponding CI *vs* Fa plot (**B**) of U87 MG cells. The concentration of the X-axis represents the molar concentration of TMZ, in addition to the MCyp group representing the Cyp concentration. Data are presented as the mean ± SD (*n* = 6).

The inhibition effect of the combination of TMZ and MCyp was also assessed in U87 MG cells which were treated with a range of ratios and concentrations of MCyp and TMZ for 72 h. Combination index (CI) was calculated according to Chou-Talalay mathematics model using CompuSyn software (ComboSyn Inc, Paranmus, NJ, USA). The CI values can be used as a judgement to the interactions of the two drugs. When CI > 1, the drugs show antagonistic action. CI = 1 means addictive action. Synergy, as indicated by a CI < 1, is assumed to be induced over a wide range of drug ratios. When the CI gets smaller, the synergistic effect appears to be stronger. For the combination of MCyp and TMZ, the calculated CI fell between 0.1 and 0.3 over a wide range of drug ratios from CI *vs* Fa curve (Figure 7). This result suggested a strong synergistic effect of Cyp on TMZ. Due to their different anti-tumor mechanisms, an ideal scenario is reached by the two-drug combination [37]. The combination also verified the enhancement of Cyp to TMZ cytotoxicity, which suggested that the two drugs may be reduced to get a more ideal result in glioma treatment.

The combination effect of the TMZ with MCyp for bulk tumor cells is quite important for determining the drug ratio in clinical therapy. It can be concluded from the IC_50_ of the MCyp and TMZ in the synergistic assay (Table 1) that MCyp and TMZ reached best synergistic effect at the molar ratio of 1:20. According to the data of synergistic study of the two drugs, we used the molar ratio of MCyp and TMZ at 1:20 for the further study.

**Table 1:** IC_50_ of MCyp and TMZ in the synergistic assay against U87 MG cells

Drug and ratio	Dose of MCyp (μM)	Dose of TMZ (μM)
Cyp	12.71	---
TMZ	---	1472.17
Cyp :TMZ = 1:2.5	16.25	40.64
Cyp : TMZ = 1:5	14.48	72.40
Cyp : TMZ = 1:10	11.24	112.43
Cyp : TMZ = 1:20	5.58	111.64
Cyp : TMZ = 1:40	8.39	335.60
Cyp : TMZ = 1:80	6.89	550.89

### Inhibition of tumor spheroids of the combination of TMZ and Cyp

**Figure 8:**
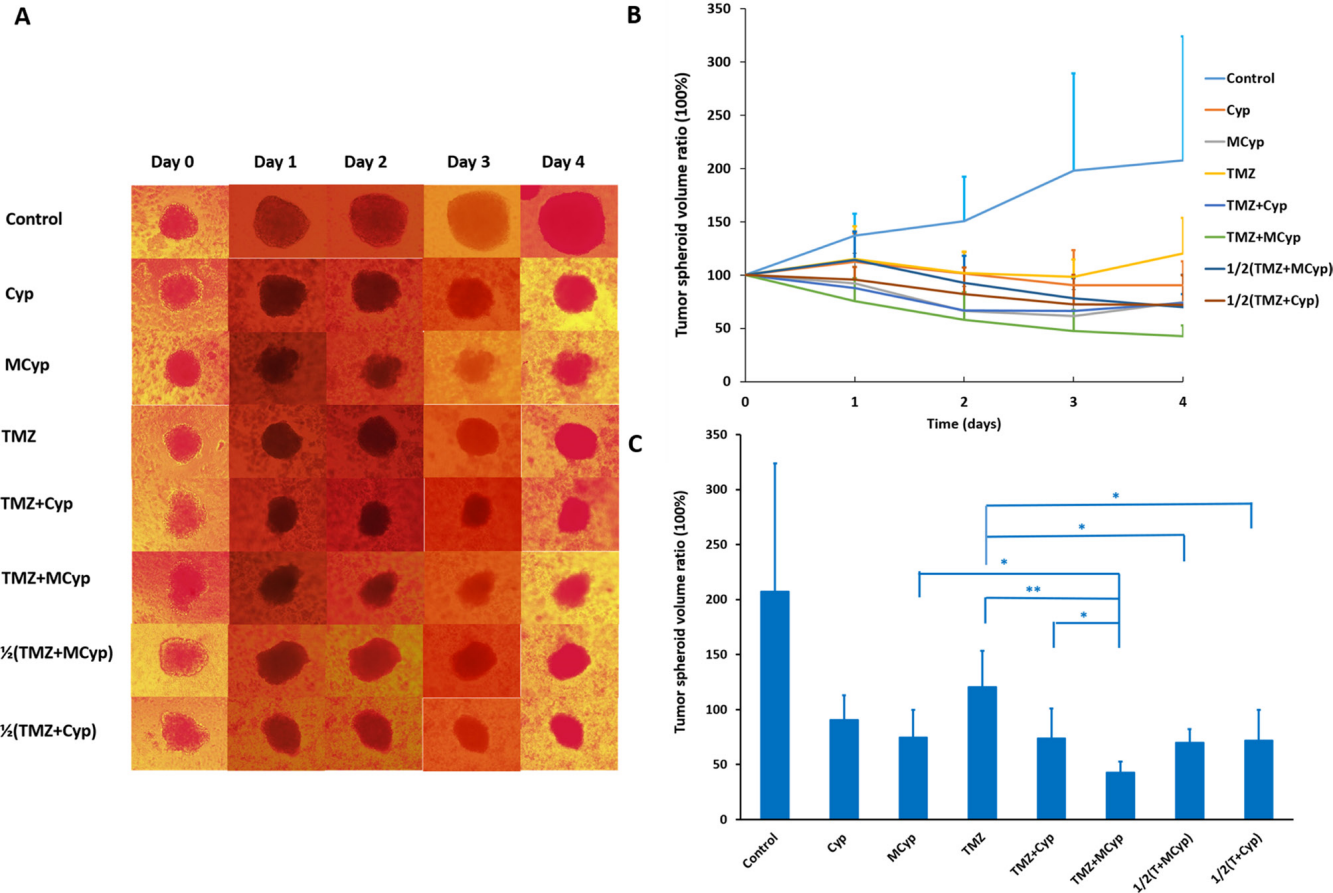
Inverted microscope photographs on Day 0~4 of U87 MG tumor spheroids after exposed to different formulations (**A**) The inhibitory effect on the growth of U87 MG spheroids after the application of drugs (**B**). Tumor volume of the fourth day compared to the initial volume of the spheroids (**C**). Data are presented as the mean ± SD (*n* = 6). 1/2(T+MCyp) and 1/2(T+Cyp) represent 1/2(TMZ+MCyp) and 1/2(TMZ+Cyp), respectively. (C)*P < 0.05; **P < 0.001.

In the process of solid tumor treated by chemotherapy, drugs need to be able to permeate and accumulate deep into the tumor to kill cancer cells, especially drug-resistant cells. Therefore, we conducted the assay of tumor spheroids to study the permeability difference between Cyp solution and MCyp, which imitated the environment that drugs encounter *in vivo* [38]. Meanwhile, the combination effect of the Cyp and TMZ was also assessed for spheroids. We assumed TMZ could kill most of the cells on the edge of spheroids and MCyp could permeate the spheroids deeply. The combination of TMZ and MCyp could inhibit the growth of spheroids more efficiently. Inhibition rate of the combination therapy was analyzed through monitoring the diameter of the tumor spheroids, with the final concentration of Cyp being 150 μM and the concentration of TMZ being 3000 μM. As the combination of MCyp and TMZ got better antitumor effect than TMZ alone, whether the combination could reduce the dosage of the two drugs is unclear. So, half dosage (75 μM for Cyp or MCyp and 1500 μM for TMZ) of the combination group was also given to the spheroids, named as 1/2(TMZ+MCyp) and 1/2(TMZ+Cyp), respectively. As it is shown in Figure 8A, after exposure to drugs, all spheroids growth rates were inhibited compared to the control (PBS treatment) and the edge of the spheroids got obscure and dark, which suggested the cells on the surface of the spheroids died after the administration. The volume ratios of the spheroids on the fourth day comparing to day 0 are 207.45%, 90.42%, 74.53%, 120.42%, 73.84%, 42.72%, 71.82% and 68.98% for Control, Cyp, MCyp, TMZ, TMZ+Cyp, TMZ+MCyp, 1/2(TMZ+Cyp) and 1/2(TMZ+MCyp), respectively (Figure 8B, 8C). MCyp inhibited the growth of U87 MG spheroids much stronger than Cyp which demonstrated that MCyp permeated the spheroids much more than Cyp solution or with lasting effect beyond Cyp solution. The spheroids in given combined administration groups got smaller and which demonstrated the more damage of the tumor cells. We suspected that with the addition of TMZ, most cells were killed at the edge of the spheroids, which facilitated the permeation-killing cycle of MCyp, followed by inhibition the deeper cells especially the drug-resistant cells. The half dosage of the Cyp and TMZ in the combination groups were also notably inhibited to a similar effect of MCyp of 75 μM and more effective than TMZ of 1500 μM, which suggested that the efficacy can also be maintained at a reduced dose and the side effects may also be reduced in clinical. In conclusion, combination therapy of TMZ with MCyp produced the most significant reduction in the tumor spheroids volume, and the dosage of the drugs can be reduced.

**Figure 9:**
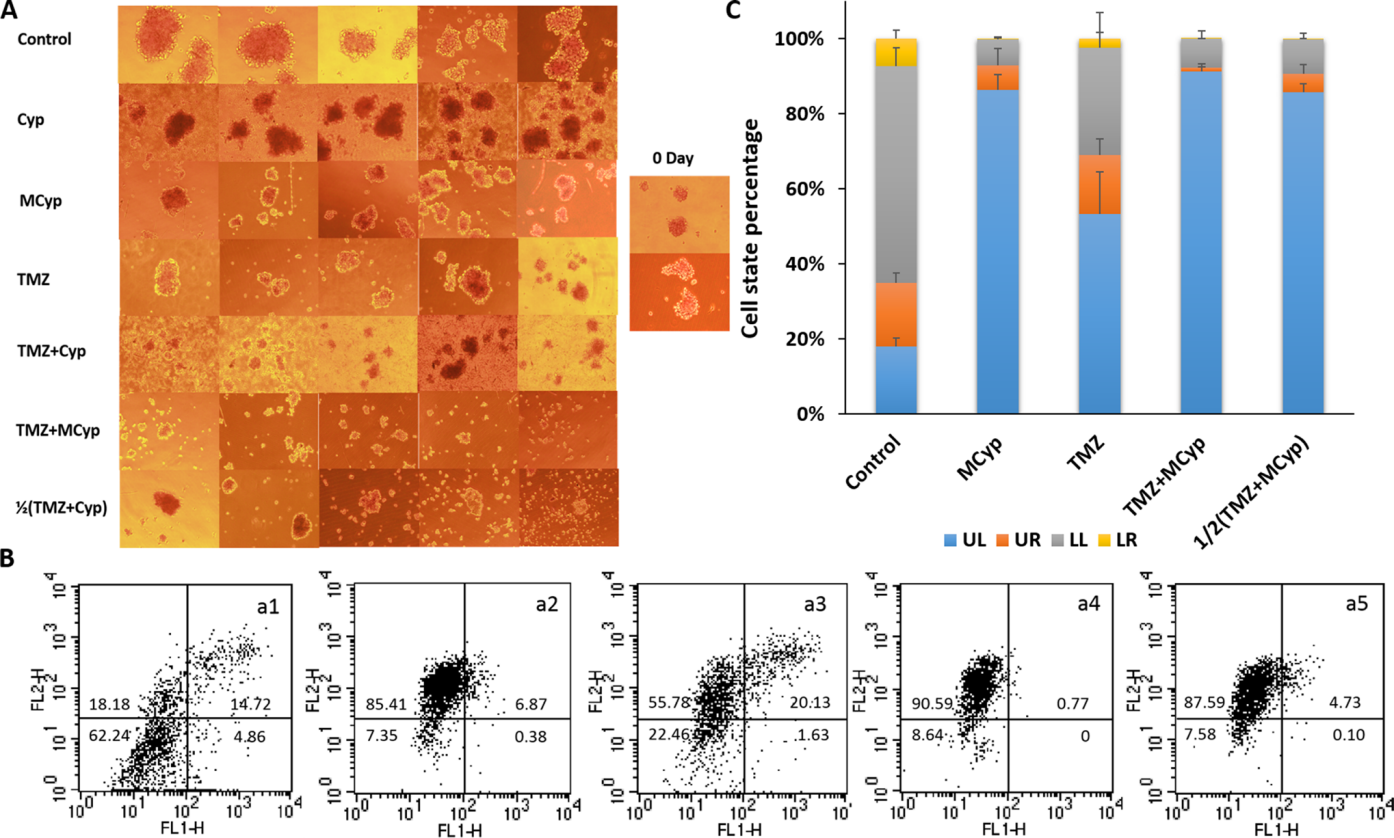
The representative pictures of the DBTRG-05MG neurospheres after treated with of PBS, Cyp solution, MCyp, TMZ, TMZ+Cyp, TMZ+MCyp, and 1/2(TMZ+MCyp) (**A**) Typical apoptosis profiles (**B**) and quantitative analysis (**C**) of the collected cells from the neurospheres after incubated with control (a1), MCyp (a2), TMZ (a3), TMZ+MCyp (a4), 1/2(TMZ+MCyp) (a5). LL, UL, LR and UR quadrant represents viable cells, necrotic cells, early and late apoptotic cells, respectively. Data are presented as the mean ± SD (*n* = 3).

DBTRG-05MG cells could form tumor spheroids-like neurospheres when cultured in neurosphere medium. After cultivation of 8 days, neurospheres were approximately 200~300 μm in diameter and labeled as Day 0. Then PBS, Cyp, MCyp, TMZ, TMZ+Cyp, and TMZ+MCyp, 1/2(TMZ+Cyp) were given. The final concentration of Cyp was 300 μM and the concentration of TMZ was 6000 μM and 1/2(TMZ+Cyp) means half dosage (150 μM for Cyp and 3000 μM for TMZ) of the combination group. Pictures were taken on Day 6 after the administration and five representative fields of each group were shown (Figure 9A). In the control group, neurospheres rapidly grew, the edge of the neurospheres seemed clear and the density remained moderate compared to the other groups. The group of Cyp solution and TMZ presented obviously smaller neurospheres. Besides, the number of the neurospheres in the Cyp solution group appeared to be much more than in the other groups, which may suggest that Cyp suppresses neurospheres in a weaker role. As to MCyp group, neurospheres got even smaller than Cyp solution group and the density in the culture medium became lower. When treated with TMZ+Cyp, there were only several neurospheres existed in the medium and a large amount of cells were suspending in the medium. The phenomenon got more obvious when MCyp and TMZ were given together, which seemed like there were no live cells in the medium, let alone neurospheres. Surprisingly, the treatment of 1/2(TMZ+Cyp) is more effective in inhibiting the growth of neurospheres than large dosage of MCyp or TMZ alone. In addition, the neurospheres of the Cyp solution group seemed darker than those without Cyp solution, it was caused by the Cyp crystal absorbed on the cell surface due to the poor solubility.

To further study the state of the cells in neurospheres, cells were collected and apoptosis assay was conducted (Figure 9B, 9C). The percentage of necrotic cells was only about 17.95% in the control group. As for MCyp, TMZ, TMZ+MCyp, and 1/2(TMZ+MCyp), the necrotic cells rate was about 86.42%, 54.34%, 91.39%, 85.84%, respectively. What is clear from the data is that MCyp gave an excellent performance in inhibiting neurospheres formation and resulted in the low viable cells. Combination therapy of TMZ and Cyp enhanced apoptosis of the cells inducted by the drugs alone and the dosage could be reduced to get equivalent efficacy.

### Cyp down-regulated the Gli1 level in U87 MG cells

**Figure 10:**
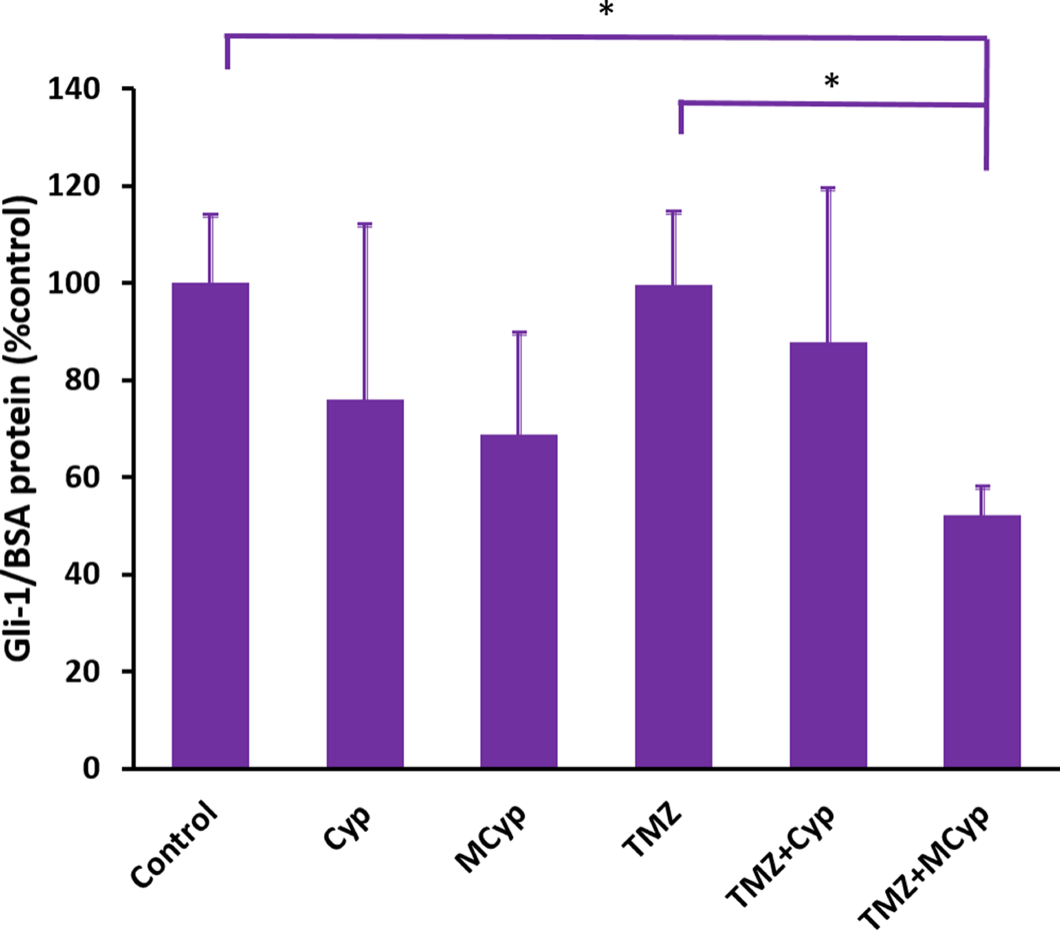
Gli1 protein level of U87 MG cells after treatment of PBS (as control), Cyp solution, MCyp, TMZ, TMZ+Cyp, and TMZ+MCyp The final concentration of the Cyp and TMZ are 30 μM and 600 μM, respectively. Data are presented as the mean ± SD (*n* = 3).

As an antagonist of Shh, Cyp should down-regulate the expression of downstream proteins of Shh pathway. Meanwhile, TMZ is an alkylation drug which would not influence the Shh pathway. To evaluate the response in protein level of Shh pathway, ELISA was conducted to analyze the expression of Gli1 in U87 MG cells after different formulations of drugs were given for 24 h. As shown in Figure 10, compared with the control group, Gli1 protein in cells treated with Cyp solution and MCyp were down-regulated to 75.94% and 68.74%, while the group of MCyp seemed to be more effective in the result. When cells were treated with TMZ, Gli1 protein was 99.64% compared to the control group, which demonstrated TMZ had no influence on Gli1 level as expected. However, combination treatment of the TMZ with Cyp solution and MCyp both down-regulated the Gli1 level to 82.73% and 52.13%, respectively. Cells treated with TMZ and MCyp together exhibited prominent down-regulation compared to the control group and TMZ alone group (*P* < 0.05). Therefore, MCyp not only influenced the pharmacological effect of Cyp, but also enhanced its effect. What's more, TMZ+MCyp functioned as the most powerful group in the inhibition of Gli1 protein, which could prove to be promising in the inhibition of Shh pathway.

## DISCUSSION

As the most effective first-line drugs in clinical treatment of glioma, TMZ is very effective in eliminating tumor parenchyma as well as being able to easily cross the BBB. It is essential in killing bulk tumor and can be used even when MGMT expression is high. Therefore, we chose TMZ as the primary drug for the treatment. However, TMZ is unable to solve the problem of glioma recurrence due to the existence of CSC, although only a small percentage of cells in solid tumor are tumor stem cells.

The tumorigenicity of GBM and its CSC are associated with Shh high expression. Shh blockage could lead to the apoptosis and death of CSC [36]. Therefore, the Shh pathway inhibitor Cyp was used as adjuvant therapy for CSC to prevent recurrence of glioma.

As Cyp has poor solubility, our goal here is to improve Cyp's solubility and combine it with existing clinical treatment to efficiently cure glioma. The encapsulation of Cyp into PEG-PLA micelles, which possess a small particle size about 30 nm, improved the drug solubility. With its preferred small size and solubility, MCyp is able to enter the cells, permeate deep into the tumor parenchyma and accumulate effectively.

As the most characterized transcription factor associated with the activation of Shh target genes, Gli1 protein expression level could be used as an indicator of the activation of Shh pathway [39]. When retrospecting the MTT results, MCyp strengthened the Gli1 attenuation of Cyp as expected, which explained why MCyp inhibited the growth of the cells more effectively than Cyp. Meanwhile, Shh regulates the stemness of glioma stem cells. Through Shh pathway blockage, colony formation was inhibited, which suggested a reduction in the self-renewal ability of CSC. All these results showed that MCyp was more promising than Cyp solution in inhibiting both proliferation and clonogenity of GBM cells. Encapsulation of Cyp to micelles guarantees its uptake into the cells, which improves the efficacy even further.

What's more, the blockage of Shh pathway also leads to the decrease of CD133, which may benefit the patients. The Shh pathway has a positive correlation with CD133^+^ expression in glioma CSC [40]. From a clinical perspective, CD133 is a significant indicator for patients with poor prognosis and high glioma grade [41]. Cyp was proposed to inhibit the stem cell-like behavior through Shh inhibition and therefore attenuate the CD133^+^ expression. Thus, the suppression of clonogenity of GBM cells by MCyp results in the loss of self-renewal properties of stem cells, which is promising in the treatment of cancer stem cell-like cells.

The diffuse migration property of the glioma is the major obstacle in glioma therapy. Surgical resection, chemotherapy or radiation therapy can not prevent glioma cells from escaping and forming new tumor nodules [42]. To increase the life expectancy of patients, not only the cytotoxicity of the drug, but also the elimination of infiltrative property of glioma are crucial. MCyp and Cyp solution both exhibit great potential in preventing the migration of glioma cells. The greater potential of MCyp in preventing cell migration was attributed to the Shh pathway blockage by MCyp. Therefore, the formulation of Cyp (MCyp) appears to be superior than Cyp in all of the pharmacological effects.

On the other hand, the combination of TMZ and MCyp down-regulated the expressions of Shh protein more prominently, which may explain why TMZ showed additive effects in U87 MG and DBTRG-05MG cells cytotoxicity assay with Shh pathway inhibitors. The results in spheroids and neurospheres inhibition experiments showed that the Shh interdiction enhanced the cytotoxicity of TMZ in diminishing spheroids volume and neurospheres number. We reasoned that the beneficial effect of MCyp in combination with TMZ is associated with the blockade of Gli1 activity.

It has been proven that cells with high CD133 expression are located at the central part of glioma and are more resistant to TMZ compared to the cells at peripheral layer [43]. The disappearance of neurospheres in the culture medium and the high apoptosis percentage of the cells suggested its inability of self-replication, which suggested tumor recurrence caused by CSC may be suppressed. The disappearance of spheroids in the combination group may be explained by the treatment of two drugs: TMZ killed most of the cells at the peripheral layer and the drug-resistant cells (mostly CSC) were killed by Cyp, which brought about the death of the cells and termination of the spheroids.

When Cyp was combined with Smo protein, there would be a reduction in downstream genes and proteins. The harmful roles of the downstream proteins would be prevented as Shh signaling pathway is aberrant in glioma. In our results, the decline of Gli1 led to significant reduction of tumor cells’ self-renewal, viability and migration ability. The addition of TMZ enhanced the down-regulation of Gli1, and had synergistic inhibitory effect on cell viability, tumor spheroids growth and neurospheres formation. The confirmed mechanism of Cyp and combination therapy displayed promising strategy for GBM treatment.

In sum, through Gli1 down-regulation, MCyp yielded better performance than Cyp solution in inhibiting cell proliferation, CSC and cell invasion, which could reduce the escape of glioma cells from chemotherapy. The combination of TMZ and MCyp reached notable synergistic effect in cytotoxicity and anti-spheroids apoptosis induction through the enhanced blockage of Shh pathway. So we propose to use TMZ to kill tumor parenchyma and MCyp as the CSC inhibitor for avoiding tumor recurrence. By working in different pathway, the two-drug solution appears to be a promising strategy for future clinical study and application.

## MATERIALS AND METHODS

### Materials

PEG_2000_-PLA_2000_ copolymer was purchased from Sigma-Aldrich (St. Louis, MO, USA). Cyclopamine (Cyp) was purchased from Nanjing Spring & Autumn Biological Engineering Co., Ltd. (Nanjing, China). Cyp solution was prepared by dissolving Cyp in DMSO and then diluted with phosphate buffered saline (PBS, pH 7.4), which the DMSO concentration is lower than 1%. All other chemicals were analytical or high performance liquid chromatography (HPLC) grade.

U87 MG cells (human glioblastoma cells) were purchased from the Institute of Basic Medical Science at the Chinese Academy of Medical Sciences (Beijing, China). DBTRG-05MG cells (human glioblastoma cells) were purchased from Guangzhou Biscien Biotechnology Co., Ltd. (Guangzhou, China). RPMI-1640 medium, modified eagle medium (MEM), Dulbecco's Modified Eagle Medium (DMEM), penicillin-streptomycin and trypsin were obtained from Macgene Technology (Beijing, China).

### Cells culture

U87 MG and DBTRG-05MG cells were grown in MEM medium or in RPMI-1640 medium, respectively, supplemented with 10% fetal bovine serum (GIBCO, USA), 100 IU/mL penicillin and 100 μg/mL streptomycin. All cells were maintained in a 37°C humidified incubator with a 5% CO_2_ atmosphere. Neurosphere (NS)-forming medium is consisted of DMEM/F12 (1:1), B27, 200 U/mL penicillin/streptomycin, 20 ng/mL Epidermal Growth Factor (EGF) and 20 ng/mL basic Fibroblast Growth Factor (bFGF) (Macgene Technology, Beijing, China).

### Measurement of critical micelle concentration

The critical micelle concentration (CMC) of PEG-PLA was measured similar to the reported fluorescence method using pyrene [44]. The pyrene acetone solution (0.1 mg/mL) was first added (50 μL) to test tubes. After complete evaporation of acetone overnight, different concentrations of copolymer solutions were added to the tubes. Solutions were shaken at room temperature for 24 h in dark and then tested at the emission wavelength of 390 nm, with the excitation spectra scanned between 300 and 380 nm. The ratio of fluorescence intensity at 340 nm and 337 nm (I_340_/I_337_) was plotted against the negative logarithm of the concentration of the polymer. The crossing point of two linear regressions was calculated as the CMC value.

### Preparation of the PEG-PLA micelles

Cyp-loaded micelles (MCyp) were prepared according to solvent evaporation method [45]. Briefly, Cyp and PEG-PLA were co-dissolved in acetonitrile, and evaporated under vacuum at 50°C for 30 min. After addition of PBS at 50°C and ultrasonic treatment for 2 min, a clear micelles solution was obtained. Finally, the solution was filtered through a 0.22 μm membrane filter. The MCyp were characterized by a transmission electron microscope (JEM-2100F, Japan) and dynamic light scattering analysis (Malvern Zetasizer 3000, UK) at 25°C.

### Quantitative determination of Cyp

The amount of Cyp in the micelles was measured by HPLC system with a UV detector (Shimadzu Corporation, Japan). A Dikma reverse-phase C18 column (4.6 mm × 250 mm, 5 μm) was used for analysis. The mobile phase was consisted of acetonitrile and water containing 0.03% trimethylamine (85/15, v/v). Flow rate of the mobile phase was 1.0 mL/min and the detection wavelength was set at 210 nm.

To evaluate the encapsulation efficiency (EE) of Cyp micelles, MCyp solutions were destroyed by adding 9 times volume of acetonitrile. All samples were analyzed in triplicate. The EE (%) was calculated as (W_total drug_-W_free drug_)/W_total drug_ × 100%.

### Cytotoxicity study

The cytotoxicity of the Cyp and TMZ to U87 MG and DBTRG-05MG cells was assessed by the MTT assay [46]. In brief, cells in exponential growth state were seeded at a density of 4000 cells/well in 96-well plates. After 24 h of incubation, cells were exposed to various concentrations of Cyp, MCyp, blank micelles and TMZ in a range of concentrations for 48 h or 72 h. Twenty microliter of 5 mg/mL MTT was added to each well of the plates. After incubation at 37°C for 4 h, medium was removed and 200 μL of DMSO was added to each well. The absorbance of each well was recorded at 570 nm using an iMark Microplate Reader (BioRad, USA).

### Cell clonogenic assay

Cell clonogenic assay was performed similar to the method reported [47]. To test the effect of MCyp and Cyp, cells were plated at 500 U87 MG cells or 800 DBTRG-05MG cells per 6-cm plate in growth medium for 24 h and then treated with Cyp or MCyp in a range of concentrations and incubated at 37°C until colonies were formed. At the end of the period, formed colonies were fixed with 4% paraformaldehyde solution and stained with crystal violet. Colonies contains more than 20 cells were counted. All experiments were performed in 3 replicates.

### Wound healing, cell invasion and migration inhibition assay

For wound healing assay, U87 MG or DBTRG-05MG cells were seeded in 12-well culture plates at a density of 2.5 × 10^5^ cells/well (U87 MG cells) or 2 × 10^5^ cells/well (DBTRG-05MG cells), and cells were grown with approximate 90% confluence after an incubation of 24 h at 37°C. Then the cell monolayers were wounded and washed twice with PBS, the cells were added with blank medium (as control), Cyp and MCyp for 18 h at an equivalent concentration of 5 μM Cyp at 37°C. Reparation of the wounding area was monitored under a microscope (Olympus, Japan).

For cell migration assay, U87 MG and DBTRG-05MG cells were seeded in the top insert of the 24-well transwell (pore diameter of 8 μm, polycarbonate, Corning, USA) at a density of 5 × 10^4^ cells/well in 100 μL of serum-free medium. The lower chambers were filled with 600 μL of culture medium containing 10% FBS as a chemo-attractant. Then, the cells were added with PBS (as control), blank micelles, Cyp and MCyp at a corresponding concentration of 5 μM of Cyp. After incubated for 30 h at 37°C, the cells on the upper surface of the membrane (non-invasive cells) were wiped with cotton swabs. The cells on the bottom side of the membrane were fixed with methanol and stained with crystal violet. Cells under the membrane were monitored by a microscope (Olympus, Japan).

Cell invasion assay was carried out with a density of 1 × 10^5^ cells/well similar to the migration assay described above, except that the upper chambers pre-coated with matrigel layer.

### Inhibition efficacy in tumor spheroids

Tumor spheroids were prepared with U87 MG cells using the hanging drop method similar to the previous reported [48, 49]. An agarose solution (1.5%, w/v) was prepared in serum-free MEM by heating the solution to 80°C and incubated for 30 min. Agarose solution (0.2 mL) is added to each well of a 48-well culture plate. After the solidification of the solution, each well was filled with 900 μL of culture medium. Twenty microliters of U87 MG cell suspensions containing 500 cells were suspended on the inside lid of a 48-well culture plate. The hanging drop cultures were incubated for 72 h, and then the cell aggregates were transferred into the culture medium to grow for 2–3 days to achieve an average diameter of 400 μm.

The antitumor activity of the micelles and the combination drugs on three dimension U87 MG spheroids was evaluated using a tumor spheroid inhibition assay. The U87 MG tumor spheroids were incubated with PBS (control), Cyp, MCyp, TMZ, TMZ+Cyp or TMZ+MCyp. The final concentration of Cyp was 150 μM and final concentration of TMZ was 3000 μM. The inhibition effects of the drugs are monitored by measuring the size of the tumor spheroids using an inverted phase microscope (Olympus, Japan). The major (d_max_) and minor (d_min_) diameters of each spheroid were recorded, and the spheroid volume was calculated as 0.5 × d_max_ × d_min_^2^.

### Cell apoptopsis assay against DBTRG-05MG neurospheres

DBTRG-05MG were seeded in 12-well culture plates at a density of 1 × 10^5^ cells/well with the cell medium of 950 μL neuroshpheres-forming medium to form spheroids after 8 days, which spheroids are at an average diameter of 200–300 μm. Neuroshpheres-forming medium (as control), Cyp, MCyp and combinations of TMZ were added to the wells, the final concentration of Cyp was 300 μM and the final concentration of TMZ was 6000 μM. The inhibition effects of the drugs are recorded by an inverted phase microscope (Olympus, Japan).

Cell apoptosis in the neurospheres was determined by Annexin V-FITC apoptosis detection kit according to the manufacturer's protocol. Briefly, DBTRG-05MG neurospheres were harvested, dispersed into single cells and suspended in the provided binding buffer. Then 5 μL of Annexin V-FITC was added to the cell suspensions. The mixture was incubated for another 15 min at room temperature in dark, followed by adding 5 μL of PI (propidium iodide). The double-stained cells were immediately analyzed by FACScan flow cytometry with 1 × 10^4^ events counted for each sample.

### Determination of Gli1 levels by enzyme-linked immunosorbent assay

U87 MG cells were seeded in a 75 cm^2^ culture flasks at a density of 1 × 10^6^ per well in MEM medium. After an incubation of 24 h period and MEM medium containing Cyp, MCyp and combinations of TMZ was added to each well. After incubation at 37°C for 24 h, the cells are washed with PBS and collected. The amount of Gli1 in the cells was analyzed using a human zinc finger protein Gli1 (Gli1) ELISA kit (CSB-EL009499HU, CUSABIO) according to the manufacturer's instructions. Protein concentrations in the samples were estimated with BCA Protein Assay Kit according to the manufacturer's instructions.

### Statistical analysis

Either Student's *t* test or a one-way analysis of variance (ANOVA) was used to evaluate the data. A *p*-value less than 0.05 was considered statistically significant.
